# Assessment of brain two-dimensional metrics in infants born preterm at term equivalent age: Correlation of ultrasound scans with magnetic resonance imaging

**DOI:** 10.3389/fped.2022.961556

**Published:** 2022-09-20

**Authors:** Philippe Vo Van, Jonathan Beck, Hélène Meunier, Perrine Venot, Gratiella Mac Caby, Nathalie Bednarek, Gauthier Loron

**Affiliations:** ^1^Department of Neonatology, Hospices Civils de Lyon, Femme Mère Enfant Hospital, Bron, France; ^2^Department of Neonatology, Centre Hospitalier Universitaire de Reims, Reims, France; ^3^Department of Pediatric Imaging, Centre Hospitalier Universitaire de Reims, Reims, France; ^4^University of Reims Champagne-Ardenne, CReSTIC, Reims, France

**Keywords:** brain, cranial ultrasound, MRI, brain imaging, preterm, metrics

## Abstract

**Context:**

Developing brain imaging is a critical subject for infants born preterm. Impaired brain growth is correlated with poor neurological outcomes, regardless of overt brain lesions, such as hemorrhage or leukomalacia. As magnetic resonance imaging (MRI) remains a research tool for assessing regional brain volumes, two-dimensional metrics (2D metrics) provide a reliable estimation of brain structures. In neonatal intensive care, cerebral ultrasound (cUS) is routinely performed to assess brain integrity. This prospective work has compared US and MRI accuracy for the measurement of 2D brain metrics and identification of overt injuries.

**Methods:**

MRI and cUS were performed at term equivalent age (TEA) in infants born before 32 weeks of gestation (GW). Demographical data and results of serial cUS (Neonatal Intensive Care Unit [NICU]-US) performed during hospitalization were gathered from medical charts. Blinded, experienced senior doctors reviewed the scans for both standard analysis and standardized, 2D measurements. The correlation of 2D metrics and inter-/intraobserver agreements were evaluated using Pearson’s coefficient, Bland-Altman plots, and intraclass coefficient (ICC), respectively.

**Results:**

In total, 102 infants born preterm were included. The performance of “TEA-cUS and NICU-cUS” when compared to “TEA-MRI and NICU-cUS” was identical for the detection of high-grade hemorrhages and close for low-grade ones. However, TEA-MRI only detected nodular lesions of the white matter (WM). No infant presented a cerebellar infarct on imaging. Intra- and inter-observer agreements were excellent for all 2D metrics except for the corpus callosum width (CCW) and anteroposterior vermis diameter. MRI and cUS showed good to excellent correlation for brain and bones biparietal diameters, corpus callosum length (CCL), transcerebellar diameters (TCDs), and lateral ventricle diameters. Measures of CCW and vermis dimensions were poorly correlated.

**Conclusion and perspective:**

The cUS is a reliable tool to assess selected 2D measurements in the developing brain. Repetition of these metrics by serial cUS during NICU stay would allow the completion of growth charts for several brain structures. Further studies will assess whether these charts are relevant markers of neurological outcome.

## Introduction

Brain magnetic resonance imaging (MRI) has brought new insight into the understanding of preterm neuropathology, becoming a gold standard in research and routine healthcare.

Overt lesions, such as white matter (WM) impairment and intraventricular hemorrhages (IVHs), account for a large part of severe, cognitive, and motor sequelae in preterm infants (1, 2). Impaired regional brain growth that has been documented in preterm infants with or without brain injury is also known to be an independent factor of poor neurologic development (3). Segmentation of cerebral structures in three dimensions has remained a research tool until now as it requires specific, additional computational processing. Two-dimensional cerebral metrics, such as bifrontal, biparietal, and transcerebellar diameters (TCDs), are well correlated with regional brain volumes (4) and can be assessed routinely. Besides, interhemispheric distance (IHD) and subarachnoid space are representative of the cerebral spinal fluid total volume. Scoring systems combining both qualitative injuries and cerebral measures have been validated (5–7).

Serial cerebral ultrasound (cUS) screening during hospitalization is recommended by most neonatal scientific societies to depict IVH or extensive WM lesions (8, 9). To our knowledge, neither 3D nor 2D cUS is used as a routine practice to assess the size or growth of brain structures in preterm infants. Some studies have reported the feasibility of US to assess the 2D measurement of brain structures, such as corpus callosum and cerebellar vermis measurements in this population (10, 11). The aim of this prospective study was to (1) compare cUS and MRI accuracy for measurement of 14 2D measurements that included parenchymal and extraparenchymal spaces dimensions, at term equivalent age (TEA) and (2) assess cUS performance for identifying brain lesions when compared to the gold standard MRI.

## Materials and methods

### Subjects

Infants born before 32 weeks of gestation (GW) were prospectively included between June 2013 and March 2014, upon admission to the level III Neonatal Intensive Care Unit (NICU) (Institut Alix de Champagne, Reims). Exclusion criteria consisted of brain malformations, absence of healthcare coverage, and parental non-agreement. The Local Institutional Review Board approved the study protocol on 6 May 2013.

### Clinical data

Clinical data were gathered from medical charts. Antenatal steroids, birth weight, gender, Clinical Risk Index for Babies II (CRIB II) score, any need for inotropic support and ventilatory requirement at 36 weeks post-menstrual age (PMA), and use of post-natal steroids (hydrocortisone or dexamethasone) were collected. Chorioamnionitis diagnosis was based on placental bacterial and anatomopathological study and the presence of increased maternal fever or biological inflammatory syndrome (maternal C-Reactive protein superior or equal to 15 mg/100 ml). Early and late-onset sepsis were defined as a positive bacterial blood culture and a positive biological inflammatory syndrome, before and after 72 h of life, respectively. Necrotizing enterocolitis was classified according to Bell criteria.

Sequential cUS (thereafter: NICU-cUS) was performed according to the protocol of the unit: within the first 3 days of life, at the end of the first week, and then once every 2 weeks by neonatal senior doctor. cUS scans included sagittal and coronal acquisition through the anterior fontanel and mastoid acoustic window. Reports of cUS performed during hospitalization were retrospectively gathered from medical charts.

### Imaging acquisition

Cerebral MRI and cUS were performed the same day, at TEA (i.e., superior or equal to 37 PMA). All examinations were anonymized.

#### Magnetic resonance imaging acquisition

A 3 Tesla Philips Achieva System with 8 channel head coils (Philips, Best, Netherlands) was used for cerebral MRI. The examination was conducted after feeding, using an MRI-compatible incubator, without sedation being administered. Conventional MRI study included sagittal T1-weighted spin echo (3 mm thickness; repetition time (TR), 500 ms; echo time (TE), 10 ms; field of view (FOV), 200 × 200 mm; matrix, 236 × 200), axial T1-weighted spin echo (3 mm thickness; TR, 600 ms; TE, 10 ms; FOV, 190 × 150 mm; matrix, 284 × 160), 3D T1-weighted gradient echo (1 mm thickness; TR, 9 ms; TE 4.6 ms; FOV 220 × 199 mm; matrix, 250 × 220), axial T2-weighted spin echo (3 mm thickness; TR, 3,000 ms; TE, 80 ms; FOV, 180 × 130 mm; matrix, 340 × 195), coronal T2-weighted spin echo (3 mm thickness; TR, 4,600 ms; TE, 150 ms; FOV, 160 × 160 mm; matrix, 204 × 204), and axial T2-weighted gradient echo (3 mm thickness; TR, 919 ms; TE, 16 ms; FOV, 200 × 157 mm; matrix, 224 × 139; angle lever 18).

#### Magnetic resonance imaging analysis

Two senior medical doctors, a pediatric radiologist (GMM) and a neuro-neonatologist (NB), conducted their own independent reviews of the MRI scans, which were displayed through IMPAX 6.0 DICOM browser (Agfa, Belgium). They were blinded to clinical data and cUS results.

##### Qualitative magnetic resonance imaging analysis

The qualitative injury scoring system focused on WM, cerebellum, cortex, lesions, and IVH sequelae. WM and cerebellar injury were evaluated for (i) nodular or cystic, (ii) unilateral or bilateral, and (iii) focal or extensive lesions. IVH sequelae were defined according to Kidokoro’s classification (3). IVH was classified using Papile classification (12). Grade I and II IVHs were classified as minor IVH.

##### Brain metrics

**FIGURE 1:**
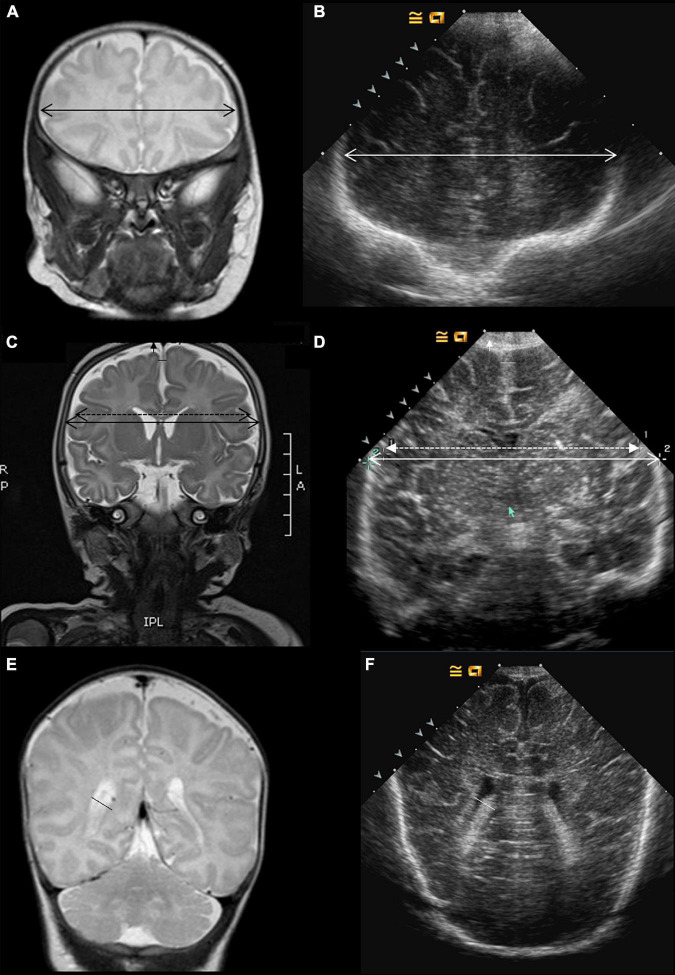
Imaging metrics protocol. MRI and ultrasound coronal views. The following metrics were compared on coronal, T2-weighted MRI sequences **(A,C,E)** and ultrasound scans **(B,D,F)**: bi-frontal diameter **(A,B)**, and lateral ventricle diameter **(E,F)**. On panels **(C,D)**, the bone parietal diameter is shown in plain double head arrow and the brain parietal diameter in the discontinued double head arrow. Measurements of extra-cerebral spaces include the subarachnoid (vertical arrowhead) and the inter-hemispheric spaces (horizontal space).

**FIGURE 2:**
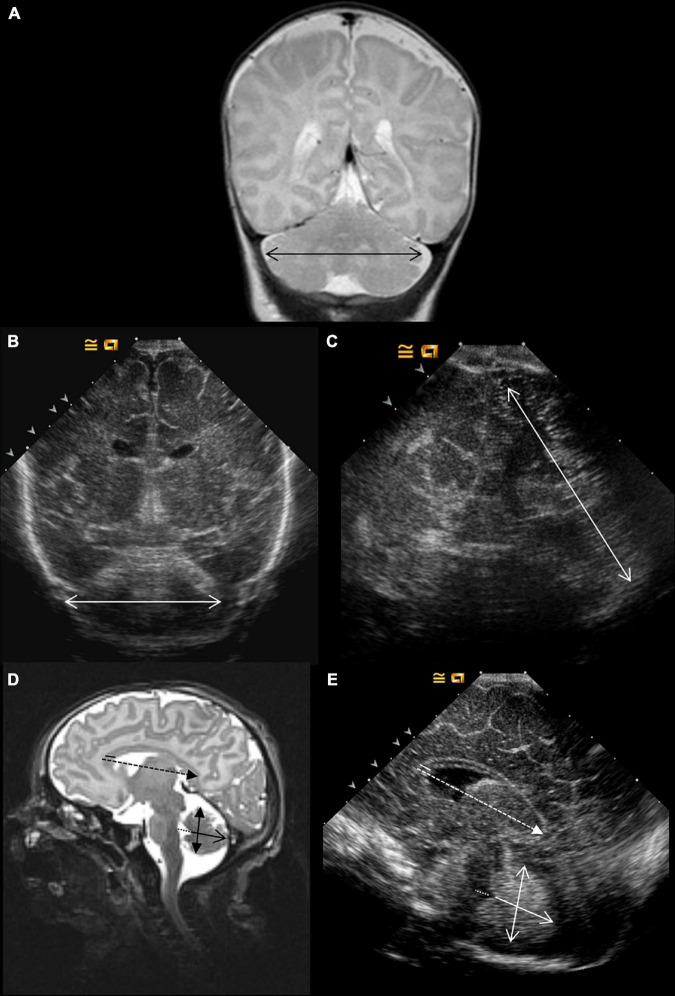
Imaging metric protocol. Coronal and sagittal views. On T2-weighted MRI, the transcerebellar diameter is measured on a coronal view **(A)**. Two acoustic windows were compared on ultrasound: coronal **(B)** and mastoid planes **(C)**. On T2-weighted MRI **(D)** and ultrasound, sagittal view **(E)** were assessed the corpus callosum length (discontinued arrow) and width (plain line), the anteroposterior vermis diameter (plain arrow) and height (double head arrow), and the fourth ventricle diameter (discontinued line).

In total, 14 variables were assessed (Figures 1, 2) following previously published methods (3, 4). Tissue metrics included bifrontal diameter (BFD), brain and bone biparietal diameter (BPD), TCD, CCL, corpus callosum width (CCW), and anteroposterior diameter and height of vermis (APVD). Fluids metrics comprised pericerebral spaces (left and right subarachnoid spaces, IHD) and intracerebral spaces (left and right lateral ventricles, fourth ventricle diameters).

#### Ultrasound acquisition

Cerebral US was performed on the same day as the MRI at TEA by PVV. cUS acquisition and analysis were blinded to MRI results and clinical data. Images were recorded using the Sequoia Ultrasound System (Siemens, Erlangen, Germany) with 8 and 12 MHz probes.

Conventional imaging protocol for qualitative analysis included five coronal views in the plane of (1) the orbits, (2) the third ventricle, (3) the fourth ventricle, (4) the bodies of lateral ventricles, and (5) the occipital lobe, and five sagittal-parasagittal views depicting (1) midline, (2) left lateral ventricle, (3) left paraventricular WM, (4) right ventricle, and (5) right paraventricular WM. All those images were acquired through the anterior fontanel.

**FIGURE 3:**
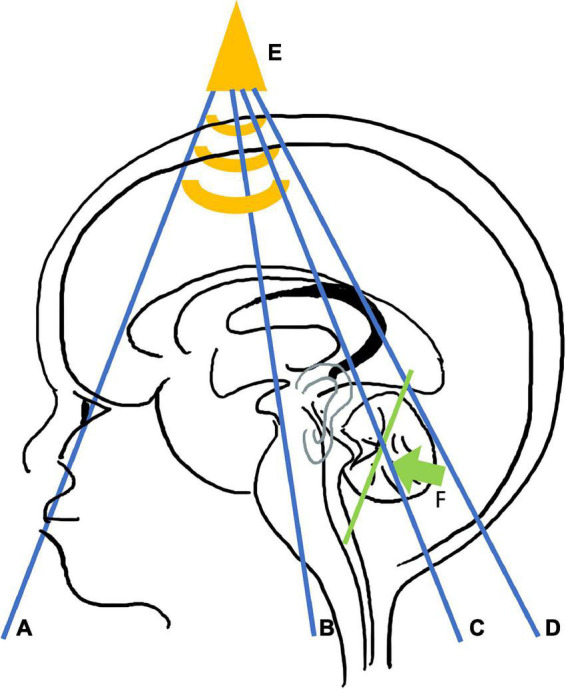
Cerebral ultrasound metrics protocol. Coronal views are represented in blue: plane **(A)** crosses the frontal lobe, just in front of corpus callosum. Plane **(B)** visualizes the parietal lobes, the temporal lobes, the third ventricle, and anterior part of the brainstem. Plane **(C)** passes through the lateral ventricles and the cerebellum at the location of its maximum width, just behind the fourth ventricle. Plane **(D)** shows the posterior horn of the lateral ventricles and choroid plexus. Bi frontal diameter is measured through plane **(A)** (Figure 1B); bone and brain parietal diameters, subarachnoidian, and inter-hemispheric spaces through plane **(B)** (Figure 1D), transcerebellar diameter through plane **(C)** (Figure 2B), and the lateral ventricle diameters through plane **(D)** (Figure 1F). Sagittal view **(E)** is displayed in yellow. The corpus callosum length and width, and the vermis anteroposterior diameter and length are assessed per this view (Figure 2E). The posterior mastoid view **(F)** is illustrated in green. The transducer is placed perpendicular to the mastoid behind the ear in order to get a coronal plane of the vermis. It allows measurement of the transcerebellar diameter (Figure 2C).

Acquisition of scans followed the protocol described in Figures 1–3. It comprises four coronal views (Plane A at the level of the orbits just in front of the corpus callosum; Plane B covering the temporal lobes, the parietal lobes, the third ventricle, and the anterior part of the brainstem; Plane C depicting the lateral ventricles and the cerebellum at its maximum width, just behind the fourth ventricle; and Plan D displaying the lateral ventricles posterior) and one midline, sagittal view passing through the anterior fontanel showing the corpus callosum and the vermis. An additional view of the cerebellum through the mastoid was recorded.

#### Ultrasound imaging analysis

A senior neonatologist (PVV) reviewed the cUS and performed the 14 measurements (Figures 1–3). A second senior neonatologist (NB) independently reviewed a set of 30 examinations for qualitative lesions and quantitative evaluation. Readers were blinded from clinical and MRI data.

##### Qualitative analysis of cerebral ultrasound

The qualitative analysis assessed the presence of IVH and grade and the presence of nodular or cystic lesions of WM and cerebellum.

##### Quantitative analysis of cerebral ultrasound

Bifrontal diameter, brain and bone BPD, left and right subarachnoidian spaces and lateral ventricles, and IHD were measured from the coronal views (Figures 1, 3). CCL and CCW, fourth ventricle diameter, and APVD were measured from the sagittal views (Figures 2, 3). TCDs were measured from the coronal and mastoidian views (Figures 2, 3) (13).

### Statistical analysis

Statistical analysis was performed with Statistical Analysis R Software 3.2.5, Free Software Foundation’s GNU General Public License. Sensitivity (Se) and specificity (Sp) criteria were used to evaluate the accuracy of TEA-cUS for assessing qualitative lesions by comparison with that of TEA-MRI. The accuracy of metrics measurements by cUS when compared to MRI was evaluated based on Pearson’s correlation coefficient and Bland-Altman Plot. Inter- and intraobserver agreements were calculated with intraclass correlation coefficient (ICC) from a sample of 25 MRI and thirty cUS scans. They were analyzed independently by two experienced reviewers, each independently from the other, and clinical data. Analysis of scans for the intraobserver agreement took place 1 month apart. A threshold of 0.7 and above was considered as a good correlation for both Pearson’s correlation coefficient and ICC.

## Results

### Subjects

In total, 102 preterm infants born before 32 GW were included. The clinical characteristics of the population are detailed in Table 1. The mean gestational age at birth was 28.9 weeks (±2.1) with a mean birth weight of 1,127 g (±313). The mean PMA at cerebral imaging was 39.3 weeks (±1.7).

**TABLE 1:** Demographic data of preterm included.

Infant characteristics	*n* = 102
Gestational Age, weeks	28.9 (2.1)
Birth weight, g	1127 (313)
Sex ratio	1.04
IUGR < 10^e^ percentile	25 (24.5%)
Antenatal steroids	94 (92.1%)
Chorioamniotitis	15 (14.7%)
CRIB II score	10.5 (3.4)
Postnatal use of steroids	11 (10.8%)
Need for inotropic support	9 (8.8%)
Necroziting enterocolitis	9 (8.8%)
Early onset sepsis	18 (18.6%)
Late onset sepsis	34 (33.3%)
Need for ventilation at 36 postmenstrual age	21 (20.6%)
Postmenstrual age at MRI, weeks	39.3 (1.7)

IUGR, intrauterine growth restriction; CRIB, Clinical Risk Index for Babies. Data are expressed in mean (SD) or in number (%).

### Qualitative analysis of brain imaging

Overall, 55 patients presented one or more qualitative anomalies on imaging (Table 2). Mild IVH (IVH grade I or II) was reported in 27 patients, grade III IVH in four, and IVH plus parenchymal infarction for one (grade IV). Nine patients were diagnosed with nodular WM lesions and three with cystic WM lesions. Nodular cerebellar injuries were found in nine patients. No cerebellar infarction was observed in this population.

**TABLE 2:** Qualitative study of brain imaging.

102 patients	Overall	TEA MRI & NICU cUS	TEA US & NICU cUS	TEA MRI	TEA cUS	NICU cUS
Exams with lesion (s)	55 (54%)	52 (51%)	44 (43.1%)	49 (48%)	29 (28%)	25 (24%)
**IVH (or sequelae of)**						
Mild (grade 1 and 2)	39 (38.2%)	37 (36.3%)	33 (32.4%)	27 (26.5%)	22 (21.6%)	17 (16.6%)
Grade 3	6 (5.9%)	6 (5.9%)	6 (5.9%)	4 (3.9%)	2 (2%)	5 (4.9%)
Grade 4	1 (1%)	1 (1%)	1 (1%)	1 (1%)	1 (1%)	1 (1%)
Nodular WM lesions	9 (8.8%)	9 (8.8%)	1	9 (8.8%)	1 (1%)	0
Cystic WM lesions	3 (2.9%)	3 (2.9%)	3	3 (2.9%)	3 (2.9%)	2 (2%)
Nodular cerebellar lesions	9 (8.8%)	9 (8.8%)	0	9 (8.8%)	0	0

IVH, intraventricular hemorrhage; WM, white matter; TEA, term-equivalent age; NICU, Neonatal Intensive Care Unit; cUS, cerebral ultrasound. Data are presented as numbers (%).

The Se and Sp of TEA-cUS reached 11.1 and 100% for nodular WM lesions and 100/100% for cystic WM lesions. Sensitivity and Sp to diagnose mild IVH sequelae (I and II grades), III grade, and IV IVH were 63 and 93.3%; 50 and 100%; and 100 and 100%, respectively. No nodular cerebellar lesions were identified using US scans.

The combination of TEA-MRI and NICU-cUS identified 52 patients with qualitative anomalies, whereas the combination of NICU- and TEA-cUS identified 44 of those patients. When adding data from NICU-cUS to TEA-MRI, ten more mild IVH were identified, as well as two more grade III IVH. When adding data from serial neonatal cUS to TEA-cUS, 11 mild IVH and four grade III IVH were further identified. When NICU-cUS was combined with TEA-cUS, the rate of normal examination was decreased from 76 to 56.9%. Finally, Se values of “TEA-MRI and NICU-cUS” and “TEA-cUS and NICU-cUS” for identifying patients with qualitative anomalies were 94.5 and 80%, respectively.

### Brain metrics at term equivalent age

**FIGURE 4:**
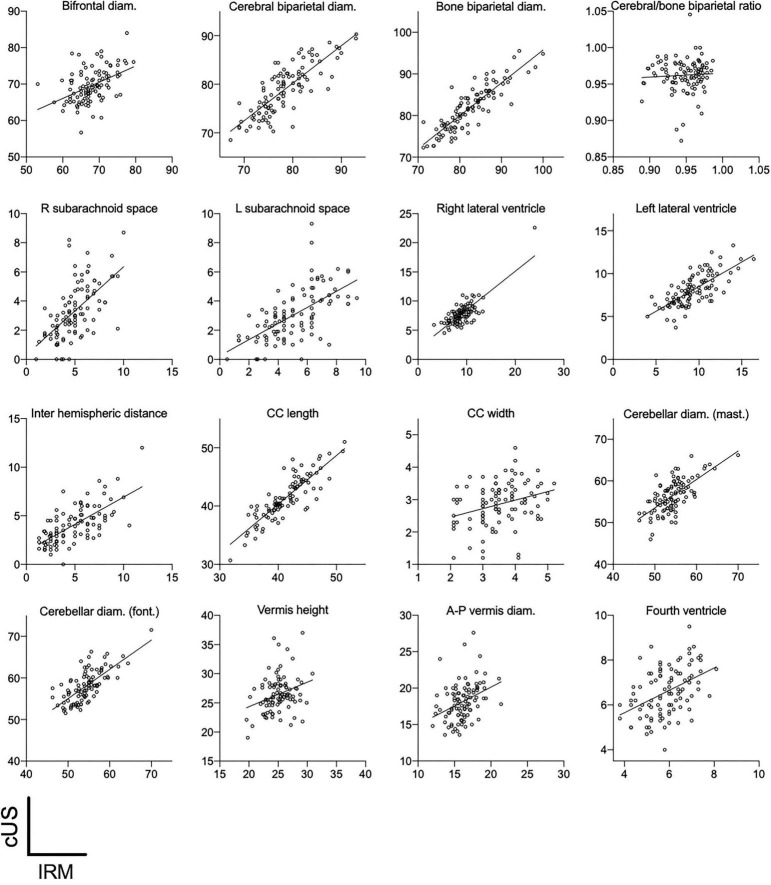
cUS vs. IRM 2D metrics. All measurements are expressed in millimeters. Diam, indicates diameter; R, right; L, left; mast, mastoid window; font, anterior fontanel window; CC, corpus callosum.

MRI and cUS brain metrics values and their correlation are reported in Table 3. Cerebral MRI and cUS show a reliable correlation (Pearson’s correlation coefficient ≥ 0.70) for brain and BPDs, length of corpus callosum, TCD through the coronal window, and diameter of lateral ventricles. The correlation of MRI and cUS measures was only moderate for brain/BPDs ratio, TCD through mastoid view, IHD, and right and left subarachnoid spaces (0.4 ≤ Pearson’s correlation coefficient < 0.7). Measures of BFD, CCW, anteroposterior vermis diameter, and fourth ventricle diameter presented low correlation (Pearson’s correlation coefficient < 0.4). Plots of correlation are reported in Figure 4, and Bland-Altman plots of MRI vs. cUS metrics are available in Supplementary Data 1.

**TABLE 3:** Comparison of metrics: MRI vs. cUS.

Total no = 102	Measurements, mm		Correlation
	MRI	Ultrasound	Pearson *r* [CI 95%]
**Bi-hemispheric diameter**			
Bifrontal diameter	68.07 (5.5)	69.6 (4.6)	0.45 [0.29–0.59]
Brain biparietal diameter	78.25 (5.4)	79.2 (4.9)	0.81 [0.73–0.87]
Bone biparietal diameter	82.57 (5.8)	82.6 (5.3)	0.87 [0.82–0.91]
Ratio brain/bone	0.95 (0.02)	0.97 (0.02)	0.57 [0.38–0.77]
**Corpus callosum**			
CC length	41.32 (3.8)	41.4 (3.8)	0.85 [0.80–0.90]
CC width	3.51 (0.8)	2.7 (0.7)	0.30 [0.12–0.47]
**Cerebellum**			
Transcerebellar diameter (CUS: Coronal window)	54.21 (3.8)	58.8 (3.8)	0.71 [0.60–0.80]
Transcerebellar diameter (cUS: Mastoid window)	54.21 (3.8)	56.7 (3.9)	0.67 [0.54–0.76]
Vermis height	25.04 (2.2)	27.0 (3.2)	0.30 [0.11–0.47]
Ant-post vermis diameter	16.16 (1.8)	18.3 (2.7)	0.36 [0.17–0.52]
**Pericerebral spaces**			
Inter-hemispheric distance	4.79 (2.4)	4.1 (2.2)	0.69 [0.57–0.78]
R sub-arachnoid space	4.96 (1.9)	3.3 (1.9)	0.60 [0.46–0.71]
L sub-arachnoid space	5.09 (1.9)	3.0 (1.8)	0.62 [0.48–0.73]
R lat ventricle diameter	8.99 (2.4)	7.4 (1.6)	0.79 [0.70–0.85]
L lat ventricle diameter	9.28 (2.3)	7.8 (2.1)	0.73 [0.61–0.80]
Fourth ventricle diameter	5.89 (0.9)	6.9 (1.1)	0.43 [0.25–0.58]

Measurements are expressed in ml presented as mean (SD). CC, corpus callosum; cUS, cerebral ultrasound; R/L right/left, ant-post, anteroposterior; lat, lateral.

### Reliability of measurements

Inter- and intraobserver ICCs for MRI and cUS are reported in Table 4.

**TABLE 4:** MRI and cUS intra- and inter-observer ICCs.

	MRI ICC		cUS ICC	
	Intra-observer	Inter-observer	Intra-observer	Inter-observer
Bi-hemispheric diameters				
Bifrontal diameter	0.968	0.780	0.966	0.969
Cerebral biparietal diameter	0.964	0.864	0.963	0.980
Bone biparietal diameter	0.979	0.962	0.963	0.952
Corpus Callosum				
Length	0.983	0.898	0.964	0.957
Width	0.737	0.547	0.623	0.625
Cerebellum				
Transcerebellar diameter (coronal window for cUS)	0.963	0.968	0.987	0.986
Transcerebellar diameter (mastoid window for cUS only)	0.963	0.968	0.832	0.860
Vermis height	0.862	0.681	0.902	0.902
Anteroposterior vermis diameter	0.871	0.772	0.719	0.713
Pericerebral spaces				
Fourth ventricle diameter	0.898	0.855	0.808	0.810
Interhemispheric distance	0.977	0.800	0.983	0.983
Right sub arachnoid space	0.984	0.710	0.913	0.931
Left sub arachnoid space	0.974	0.700	0.958	0.965
Right ventricular diameter	0.931	0.870	0.953	0.868
Left ventricular diameter	0.902	0.884	0.986	0.835

ICC, intraclass coefficient; cUS, cerebral ultrasound.

Intraobserver correlation coefficients for MRI metrics were excellent (above 0.7). Inter-observer ICC was higher than 0.7 for most of the metrics, except for CCW (0.547) and vermis height (0.681).

Intraobserver and inter-observer correlation coefficients for cUS were excellent for all metrics except the CCW (ICC 0.623 and 0.625, respectively). Unlike the MRI results, inter-observer reliability of vermis height was excellent.

## Discussion

To our knowledge, this is the first study that assesses the correlation between US and MRI 2D measurements of the brain—or “metrics”—in infants born preterm. Correlation of those metrics is good to excellent for brain and BPDs, CCL, cerebellar diameter, and most of the measures of cerebrospinal fluid (CSF) spaces. Accuracy is lesser for CCW, vermian dimensions, fourth ventricle, and BFDs. Inter-reproducibility and intraobserver reproducibility are excellent except for CCW for both techniques and vermis height for MRI inter-observer reliability only.

The qualitative analysis demonstrates that TEA-MRI depicts more nodular lesions when compared to cUS in cerebral WM and cerebellum, even when combined with results of serial cUS acquired during the NICU stay.

### Qualitative study

Moderate to severe WM lesions on MRI predict cerebral palsy with Se between 60 and 77% and Sp between 79 and 96%. They also diagnose a probability of severe neurologic impairments (involving blindness or auditive deficit) with Se and Sp between 53 and 82% and 82 and 85%, respectively (1, 5–7).

In the study’s sample, moderate to severe brain WM lesions were present in 12% of patients. Twelve patients showed peri-ventricular leukomalacia (PVL): nine with nodular WM lesions and three with cystic lesions. Four had grade III IVH and one post-hemorrhagic infarction. These results are relatively consistent with the findings of large cohorts (8).

We did not observe any cerebellar hemorrhage among the included patients, either on US imaging or MRI. In our experience, the mastoïdian acoustic window is more efficient for cerebellar imaging, compared to the transfontanellar acoustic window. This has been corroborated elsewhere (9). Cerebellar infarction, occurring especially in the first weeks of life of babies born extremely preterm, can have a very serious impact on neurological outcomes and should be screened during serial cUS (10).

Cerebral US may find 79% of major abnormalities related to cerebral palsy (i.e., cystic PVL and major IVH) with sensitivity and specificity of 76 and 95%, respectively (6, 11). Compared to susceptibility-weighted imaging MRI sequence, Se and Sp of cUS were 100 and 93% to detect grade III IVH and 66.7/100% for grade IV IVH (12). In this work, T1 and T2 sequences were found to be as efficient as susceptibility-weighted imaging sequences. The Se and Sp of cUS at TEA to diagnose grade III and IV IVHs were, respectively, 50/100 and 100/100%. Retrospectively, some images interpreted as grade III IVH on MRI were classified as a ventricular enlargement on cUS, as no sequelae of bleeding were identified.

Ultrasound had failed to identify nodular PVL; only one out of nine led to a low Se of 11%. Sensitivity and Sp of Us as compared to MRI for the diagnosis of nodular PVL were known to be low: from 26 to 38% and 85 to 96%, respectively (13, 14). This may be a limitation of technique and resolution itself.

When combining data from a sequential US performed during hospitalization with the term-equivalent US, the accuracy of an “all-ultrasound protocol” to detect IVH from grades I to III is improved. However, performance remains low for nodular lesion detection. These results must be interpreted with caution due to the retrospective collection of NICU-cUS.

Overall, TEA-brain MRI proves to be a superior means of diagnosis than TEA-cUS for a complete detection, quantification, and characterization of qualitative WM abnormalities, especially nodular lesions.

### Metrics as a surrogate of qualitative analysis

Evaluating brain growth in the preterm population is of critical interest. Premature birth disrupts brain growth independently of the development of WM lesions or IVH (15, 16). Many 3D segmentation brain MRI studies demonstrated decreased cerebral volume across many structures in infants born preterm (17, 18). These alterations persist after childhood and are firmly correlated with cognitive issues and behavioral disorders (15, 19–22). However, 3D segmentation remains unavailable in routine practice while 2D metrics provide a reliable and convenient way to obtain markers of brain growth. A strong correlation was found between BFD, BPD, and TCD and total brain and cortical gray matter and volumes (4, 23). IHD was also correlated with total CSF volume (24). Finally, enlarged lateral ventricle is a common feature of WM loss of substance (13, 14).

This study shows that cUS provides good to excellent estimation of cerebral and fluid metrics by comparison to MRI: bifrontal, biparietal, TCDs, CCL, ventricle diameters, and the IHD.

Corpus callosum is a large WM bundle involved in the inter-hemispheric communication. Premature birth impacts callosal growth and disturbs WM connectivity and the myelination process (20). Such alteration of callosal growth and microstructural disorganization has been correlated with a worse cognitive and motor outcome than a control population (20, 22, 25). In a cohort of very preterm infants <30 weeks, the majority of survivors did not have severe brain injuries but an altered biparietal diameter and IHD (24). Both altered metrics were associated with adverse neurologic outcomes at 2 years old (23, 24). An enlargement of the IHD may reflect an early-impaired cortical growth with a preserved skull growth (preserved head circumference). The consequences of the impaired cortical growth on skull growth manifest themselves at a later stage in life, therefore, head circumference at term equivalent age was not correlated with adverse outcome (26).

In the absence of CSF circulation impairment, increased lateral ventricle diameter in preterm infants is a marker of atrophy and WM loss. Ventricle enlargement is known to be correlated with pejorative cognitive and motor outcomes at 2 years old (27). Moreover, the relation with cognitive scores remained statistically strong after the exclusion of concomitant brain pathology (cystic PVL, IVH, deep gray matter, or cerebellum lesions) with a trend of alteration in a fine motor score (27).

The cerebellum is an area of particular interest in preterm infants because of its acute growth during the third trimester of the pregnancy. Cerebellar diameter at term equivalent age seems to be independently related to cognitive and motor outcomes (25, 27). This finding was also supported by the correlation between cerebellar abnormal dimensions and motor function at 7 years old (10). The cerebellar is thought to support cerebral hemisphere development with a precise topographic correlation; a cerebellar lesion may disrupt the development of cortical area. This phenomenon has been reported as a “cerebellar diaschisis” (10, 19). Cerebellar metrics were shown to be interrelated with deep GM areas whose basal ganglia and thalami receive afferences from the cerebellum (27).

In total, 14 metrics have been evaluated on MRI and cUS. The corpus callosum, parietal lobes, inter-hemispheric space, and transversal diameter of the cerebellum were the structures most accessible by US probes through the anterior or the mastoid window, which explains their high ICC scores. An anterior fontanel window was most appropriate to view supratentorial structures. The third ventricle is a straightforward landmark to spot by US. On MRI scans, it is lined up with the cochleas, which is the reference landmark described by Tich et al. to measure the biparietal diameter, IHD, and the subarachnoid spaces (4). There was greater variation in the evaluation of subarachnoid spaces although cUS allows to readily view them. One hypothesis is that the pressure of the probe on the anterior fontanel might vary the space between the subarachnoid layer and the cerebral gyrus. The acquisition of the pictures through the frontal lobe differs between MRI and US. MRI slices are acquired perpendicularly to the cerebral axis whereas the US scans through the anterior fontanel constitute a vector-based sector, which may be the cause of the discrepancy between the US and MRI measurements. Regarding the CCW, the correlation score is low, probably due to the under-5 ml size of the structure.

For some infra-tentorial metrics, US failed to get a reliable correlation with MRI. The vermis and the fourth ventricle were not outlined well enough to allow accurate measurement. The mastoid window provides better resolution and is considered the most accurate US method for the imaging of cerebellar structure as compared to anterior fontanel window (28). The study also corroborates the primacy of the mastoid window in this particular aspect, since it provides a higher ICC TCD score when compared to the standard coronal view. However, the plane in which the measurements are performed differs from that in MRI, which may explain the less compelling correlation of TCD.

In a recent work, Skiöld et al. (29) investigated the validity and prognostic value of a standardized scoring system for TEA-cUS. They compared it to a previously validated scoring system for TEA-MRI (3) and demonstrated comparable performance of TEA-cUS and TEA-MRI for the prognosis of cerebral palsy, and close for the prognosis of severe cognitive delay. The scoring system included qualitative, visual analysis, and “metrics” consisting of measurements of intra- and extra-parenchymal CSF and corpus callosum thickness. One could envision integrating the most reliable US parenchymal metrics described in the present study into such a scoring system and endeavor to further refine the performance of US imaging in establishing neurodevelopmental prognosis, especially regarding the cognitive outcome.

## Conclusion

Brain MRI constitutes the gold standard means at term equivalent age for assessing cerebral complications of prematurity. Routine score MRI includes a qualitative assessment of cerebellum, lateral ventricles, cortical folds, deep gray and WM, and quantitative measures of brain structures (24). It has been validated in different preterm populations at term equivalent age and preterm PMA (24, 30). On the other hand, cUS constitutes a useful tool in routine practice as it provides a rapid and handy means of investigation and is mastered by a vast majority of neonatologists. As the assessment of cerebral growth becomes a crucial variable to predict the neurologic outcome, this study demonstrates the feasibility of cUS to evaluate through 2D metrics impaired brain growth. Moreover, US examination can be conducted repeatedly during the hospital stay, allowing for serial assessment of brain growth.

## Data availability statement

The raw data supporting the conclusions of this article will be made available by the authors, without undue reservation.

## Ethics statement

The studies involving human participants were reviewed and approved by Reims Institutional Review Board. Written informed consent from the participants’ legal guardian/next of kin was not required to participate in this study in accordance with the national legislation and the institutional requirements.

## Author contributions

PVV, HM, PV, GM, NB, JB, and GL: conceptualization. PVV, HM, PV, GM, and NB: methodology. PVV, NB, JB, and GL: validation. PVV and GL: formal analysis and writing–original draft preparation. GL and NB: writing–review and editing. NB: supervision and project administration. All authors have read and agreed to the published version of the manuscript.
