# Supplementary figures and images for: Assessment of brain two-dimensional metrics in infants born preterm at term equivalent age: Correlation of ultrasound scans with magnetic resonance imaging
Source: Front Pediatr. 2022 Sep 20;10:961556. doi: 10.3389/fped.2022.961556 (PMC9531030; doi:10.3389/fped.2022.961556)

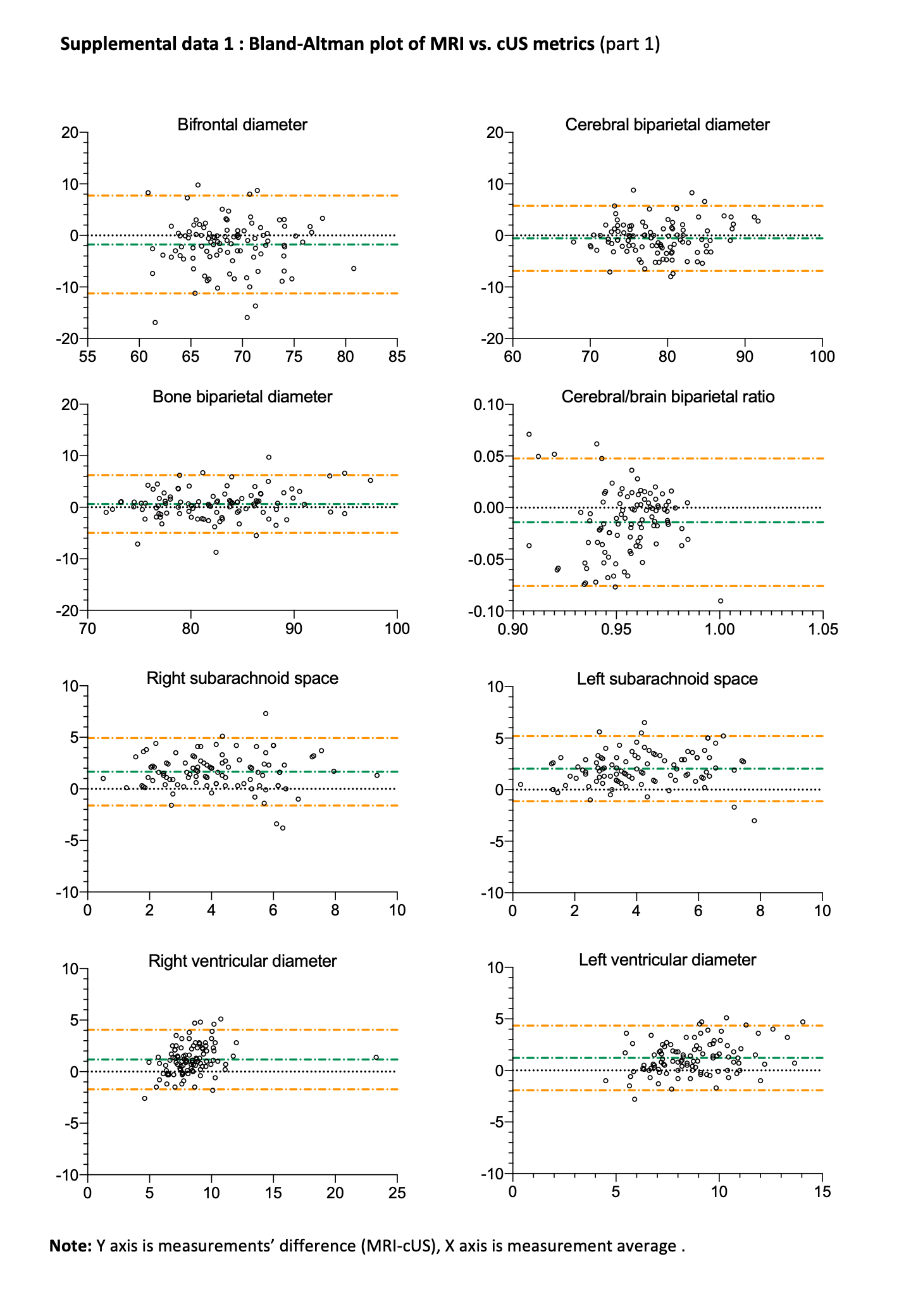

Supplement: Supplementary file 1 [file Image_1.tiff]

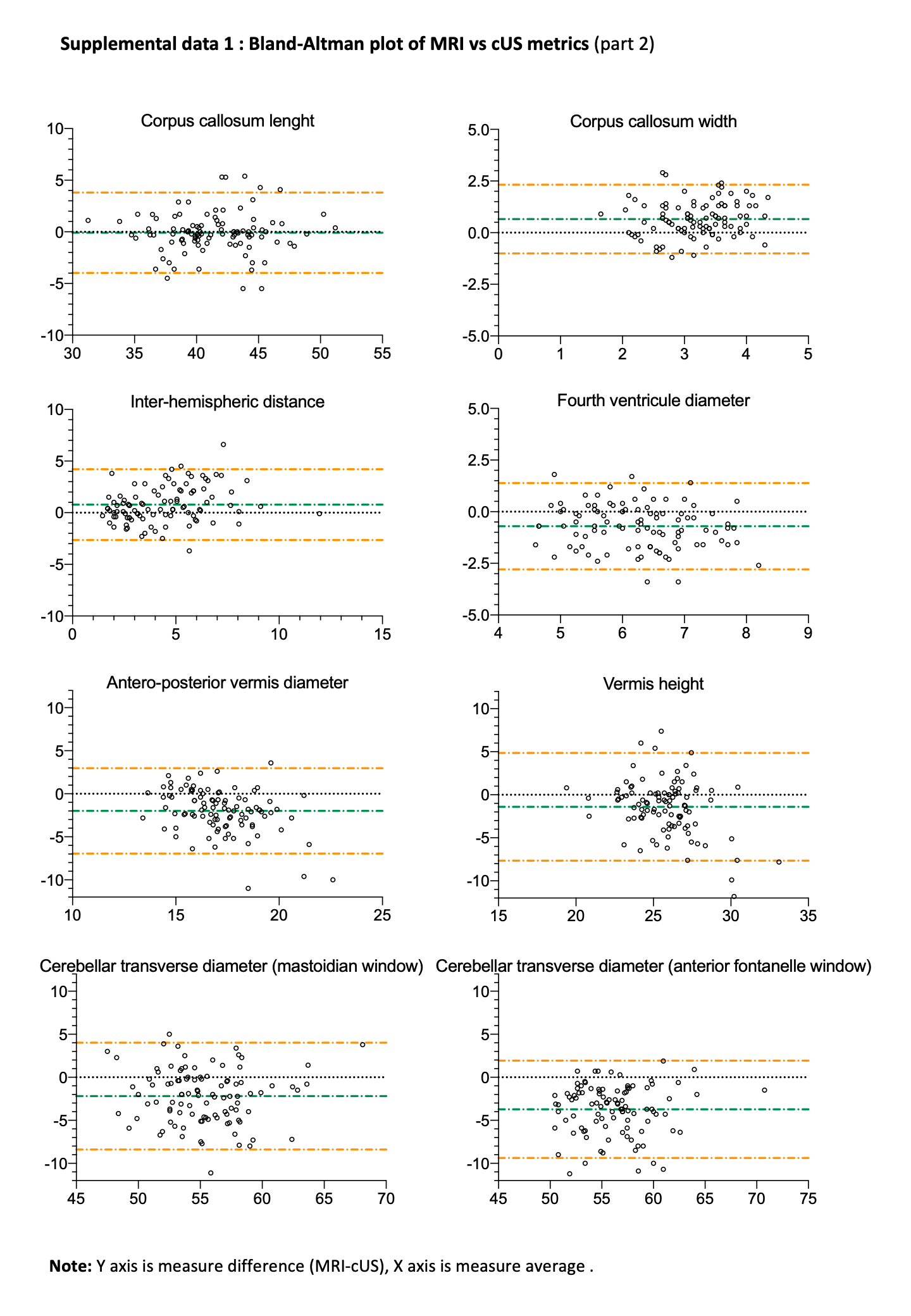

Supplement: Supplementary file 2 [file Image_2.tiff]
